# Intervention and in-hospital pharmacoterapies in octogenarian with acute coronary syndrome: a 10-year retrospective analysis of the Malaysian National Cardiovascular Database (NCVD) registry

**DOI:** 10.1186/s12877-021-02724-7

**Published:** 2022-01-04

**Authors:** Siti Z. Suki, Ahmad S. M. Zuhdi, ‘ Abqariyah A. Yahya, Nur L. Zaharan

**Affiliations:** 1 Department of Pharmacology, Universiti Malaya, 50603 Kuala Lumpur, Malaysia; 2 Cardiology Unit, Department of Medicine, Universiti Malaya, 50603 Kuala Lumpur, Malaysia; 3 Department of Social and Preventive Medicine, Universiti Malaya, 50603 Kuala Lumpur, Malaysia

**Keywords:** Cardiovascular disease, Octogenarians, Mortality, Intervention, Pharmacotherapies, Pharmacoepidemiology

## Abstract

**Background:**

Octogenarians and beyond have often been neglected in the populational study of disease despite being at the highest point of non-modifiable disease risk burden and the fastest-growing age group for the past decade. This study examined the characteristics and in-hospital management of octogenarian patients with acute coronary syndrome (ACS) in a multi-ethnic, middle-income country in South East Asia.

**Method:**

This retrospective study utilised the Malaysian National Cardiovascular Disease- ACS (NCVD-ACS) registry. Consecutive patient data of those ≥80 years old admitted with ACS at 24 participating hospitals from 2008 to 2017 (*n* = 3162) were identified. Demographics, in-hospital intervention, and evidence-based pharmacotherapies over the 10-years were examined and compared across groups of interests using the Chi-square test. Multivariate logistic regression was used to calculate the adjusted odds ratio of receiving individual therapies according to patients’ characteristics.

**Results:**

Octogenarians made up 3.8% of patients with ACS in the NCVD-ACS registry (mean age = 84, SD ± 3.6) from 2008 until 2017. The largest ethnic group was Chinese (44%). Most octogenarians (95%) have multiple cardiovascular risk factors, with hypertension (82%) being the main. Non-ST-elevation myocardial infarction (NSTEMI) predominated (38%, *p* < 0.001). Within the 10-year, there were positive increments in cardiovascular intervention and pharmacotherapies. Only 10% of octogenarians with ACS underwent percutaneous coronary intervention (PCI), the majority being STEMI patients (17.5%; *p* < 0.05). More than 80% were prescribed aspirin (91.3%) either alone or combined, dual antiplatelet therapy (DAPT) (83.3%), anticoagulants (89.7%) and statins (89.6%), while less being prescribed angiotensin-converting enzyme inhibitors/angiotensin receptor blockers (47.6%) and beta-blockers (43.0%). Men were more likely to receive PCI than women (adjusted Odds Ratio (aOR): 0.698; 95% CI: 0.490–0.993). NSTEMI (aOR = 0.402, 95% CI: 0.278–0.583) and unstable angina (UA) (aOR = 0.229, 95% CI: 0.143–0.366) were less likely to receive PCI but more likely given anticoagulants (NSTEMI, aOR = 1.543, 95% CI: 1.111–2.142; UA, aOR = 1.610, 95% CI: 1.120–2.314) than STEMI. The presence of cardiovascular risk factors and comorbidities influences management.

**Conclusion:**

Octogenarians with ACS in this country were mainly treated with cardiovascular pharmacotherapies. As the number of octogenarians with ACS will continue to increase, the country needs to embrace the increasing use of PCI in this group of patients.

**Supplementary Information:**

The online version contains supplementary material available at 10.1186/s12877-021-02724-7.

## Background

The ageing population are increasing globally, a phenomenon known as the ‘silver tsunami’. An estimated 125 million individuals reaching 80 years and older were considered octogenarians in 2018 [1]. Cardiovascular (CV) disease remains the leading cause of death in most middle-income and developing countries [2]. Age-related changes in vascular wall elasticity, coagulation and haemostatic system and endothelial functions become more apparent in this age group [3]. The prevalence of CV risk factors such as hypertension, diabetes mellitus (DM), and dyslipidaemia are also high in this age group, requiring appropriate prevention and intervention [4]. Octogenarians and beyond are often neglected when evaluating the effectiveness of intervention in clinical trials, making them poorly represented. Moreover, they are more likely on polypharmacy due to comorbidities than younger patients putting them at greater risk of drug interactions and adverse drug reactions [5]. Despite limited clinical trials and clinical issues surrounding octogenarians, those who received recommended pharmacotherapies were shown to have lower in-hospital mortality than those who did not [6].

Malaysia is a multi-ethnic, upper-middle-income country in South-East Asia with a 32 million population [7]. Malaysians aged 80 years and above are estimated to increase from 0.3 million in 2020 (0.9%) to 1.4 million (1.7%) by 2040 [8]. The prolonged lifespan is contributed by economic growth and public health development such as medical advances, better access to treatment and preventative measures [9]. CV disease remains the principal cause of death in the elderly, mainly contributed by acute coronary syndrome (ACS) [10]. There have been increases in facilities providing timely percutaneous coronary intervention (PCI) and trained cardiologists over the decades [11]. However, CV risk factors, such as DM, were also among the highest in the region [12, 13], influencing the management and outcomes of these patients [14].

Though healthcare services are available for all, demographic variation may influence patient management. Variations in intervention and pharmacotherapies for ACS patients in multi-ethnic Malaysia have previously been described [15, 16]. Less is known on the trends in the advanced age group. This study aimed to examine the 10-year trend in the characteristics of octogenarian patients with ACS, and in-patient management, focusing on cardiac intervention and evidence-based pharmacotherapies using the National Cardiovascular disease Database (NCVD) registry. The NCVD registry recorded information on patients with cardiovascular diseases from 2006 till current from hospitals across Malaysia.

## Methods

### Study population and ethical approval

This cross-sectional descriptive study is a part of research approved by the Medical Review and Ethics Committee (MREC), Malaysian Ministry of Health (MOH), with approval code of NMRR-19-4066-52,389 (IIR). MREC waived informed consent for NCVD.

Anonymised patient data were obtained from the NCVD-ACS registry, which forms part of the NCVD registry. This registry was endorsed by the Ministry of Health, Malaysia, and co-sponsored by the National Heart Association of Malaysia (NHAM) [17]. The NCVD-ACS registry recorded vital information on patients with ACS (i.e. unstable angina (UA), ST-segment elevation myocardial infarction [STEMI] and non-ST segment elevation myocardial infarction [NSTEMI]), such as demographic and clinical information and their in-hospital management, including cardiovascular interventions and medications from 24 participating hospitals across Malaysia using standardised case report form. The details of this registry have been described elsewhere [18]. This study follows the Strengthening the Reporting of Observational Studies in Epidemiology (STROBE) guidelines to ensure transparency of reporting [19].

### Patients and public involvement

There was no patient or public participation in the development of this study’s research question and outcome. All consecutive patient data from the year 2008 to 2017 were obtained retrospectively from the Malaysian NCVD-ACS registry.

### Patients characteristics

Malaysian citizens aged 80 or older (known as octogenarian) diagnosed with ACS (STEMI, NSTEMI and UA) based on the American College of Cardiology/American Heart Association (ACC/AHA) definitions [20] were identified from the NCVD registry from the year 2008 to 2017 (*n* = 3162). Patient information obtained were demographics, risk factors, and co-morbidities, summarised in Table 1. In this study, the admission of nonagenarians and centenarians are relatively low compared to octogenarians to be analyzed resprectively thus they are grouped together as octogenarians. Ethnicity and nationality were determined based on their national identification number recorded in the National Registration Department, which holds their record from birth until deceased. The major ethnicities in Malaysia are Malays, Chinese and Indians. All other minority ethnicities were categorised as ‘Others’. Patient information for each respective studied year is available in Supplementary Table 1.

**Table 1:**
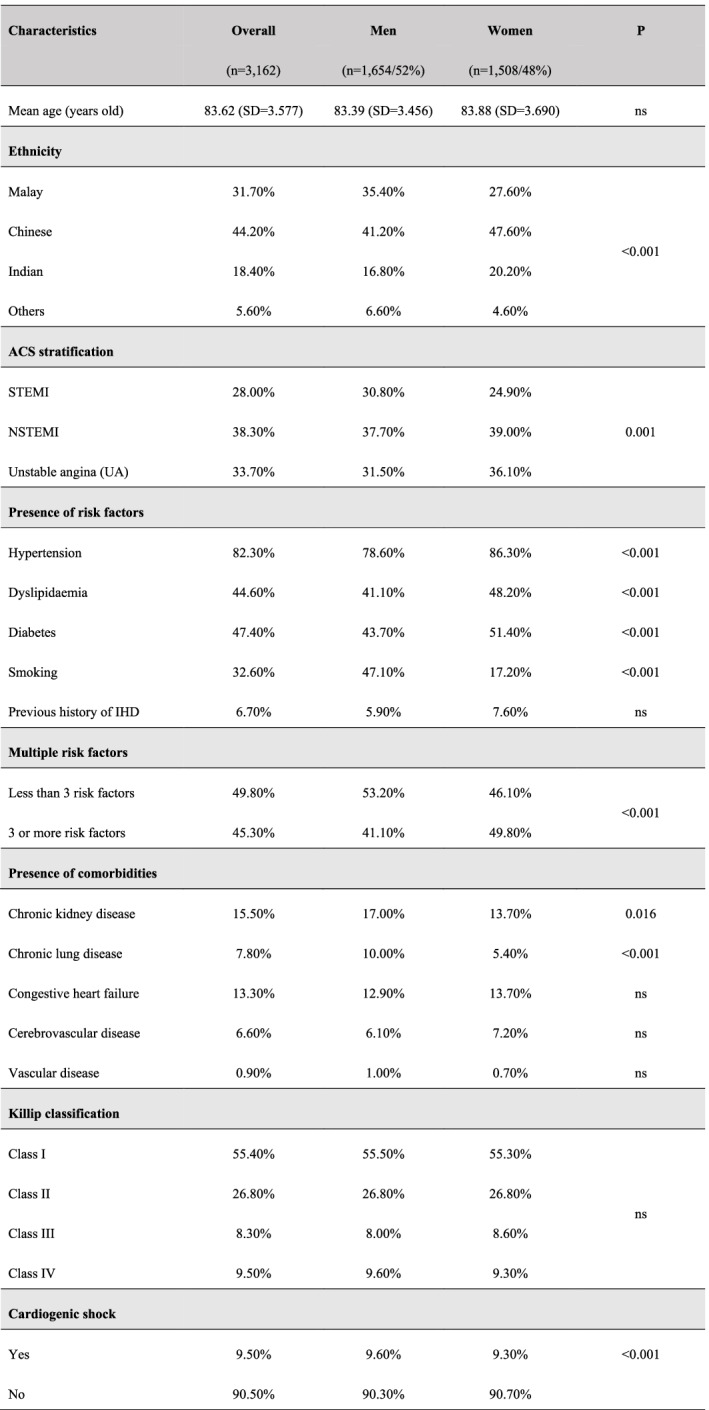
Demographics and characteristics of Malaysian octogenarians with ACS from 2008 to 2017 in the NCVD-ACS registry

Multiple risk factor *: those reported with no known risk factor are excluded. Killip Score**: Values counted from patients with STEMI and NSTEMI only. P: probability value. ns: not significant (*p* > 0.05). SD: standard deviation

### Intervention and evidence-based pharmacotherapies

In-hospital interventions included in this study were coronary artery bypass graft (CABG) and percutaneous coronary intervention (PCI), both emergency and non-emergency. Further details of the PCI intervention are available in the Malaysian NCVD-PCI registry [21]. The pharmacotherapies included were [1] aspirin, either monotherapy or combined [2] dual antiplatelet therapy (DAPT), [3] anticoagulants including unfractionated heparin (heparin), low-molecular-weight heparin (LMWH) and fondaparinux, [4] lipid-lowering agents (statins only), [5] angiotensin-converting enzyme inhibitors (ACEIs) and angiotensin receptor blockers (ARBs), and [6] beta-blockers.

### Statistical analysis

Continuous variables (age) are presented as mean ± standard deviation (SD), while categorical variables are presented as frequencies and percentages. Patient characteristics were compared across gender using Student’s t-test and chi-square test as appropriate. Choice of in-hospital intervention and evidence-based pharmacotherapies were analysed as cumulative, comparing the different ACS stratum using the chi-square test. The trends over the years were analysed using the linear trend test. Variations of in-hospital management for different groups of interest according to demographics, risk factors and co-morbidities were analysed using multivariate logistic regression, presented as adjusted odds ratio (aOR) with a 95% confidence interval (CI). The choice of covariates was based on factors influencing ACS management in the literature and included gender, ethnicity, types of ACS, risk factors and co-morbidities [22]. A probability (*p*) value of less than 0.05 is considered statistically significant. Statistical analysis was performed using Statistical Package for Social Science (SPSS) software (version 26.0, SPSS, Inc., Chicago, IL, USA).

## Results

**Fig. 1:**
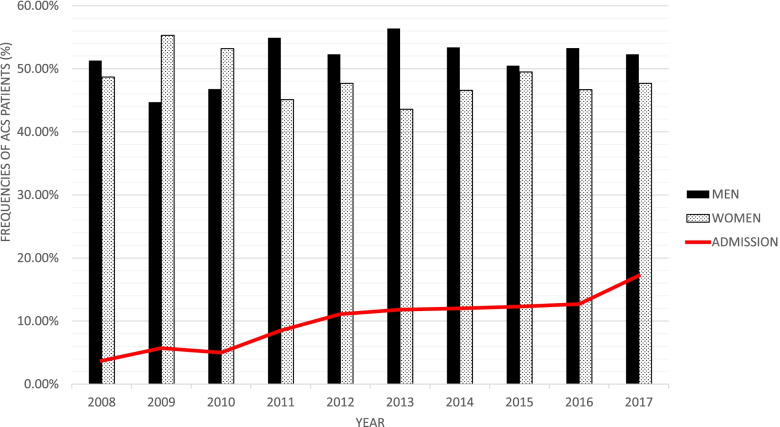
Frequencies of admission for octogenarian patients with ACS over total admission of patients with ACS in the NCVD-ACS registry (2008–2017). Gender distribution for octogenarian patients with ACS in the NCVD-ACS registry (2008–2017). 10-years cumulative *N* = 3162 comprises of 1654 octogenarians men and 1508 octogenarians women

A total of 3162 octogenarians (52% men, mean age = 83 ± 3.456 years old) was admitted with ACS from 2008 to 2017. Octogenarians made up 3.8% of the total ACS patients cumulatively during the study period and have increased significantly over the years from 3.7% in 2008 to 17.2% in 2017 (*p* < 0.001) (Fig. 1). Chinese ethnicity made up most octogenarian patients with ACS (44%), followed by the Malays, Indian and other ethnicities, with significant differences in ethnic distribution across gender (Table 1). NSTEMI and UA made up more than two-thirds of ACS presentation in this group of patients with gender differences (*p* = 0.001).

Most patients (95%) already had at least one CV risk factor at presentation. Hypertension was the highest recorded risk factor at 82%, followed by DM at 47%. There were significant differences in the CV risk factors profile between gender, with women having higher rates of hypertension, DM, and dyslipidaemia (Table 1). In comparison, men had higher rates of smokers and previous history of ischaemic heart disease (IHD). Chronic kidney disease (CKD) was the commonest co-morbidities (16%), followed by congestive heart failure (13%). More than half of patients were in Killip class 1 at presentation (55%), with only 10% considered as having cardiogenic shock.

**Fig. 2:**
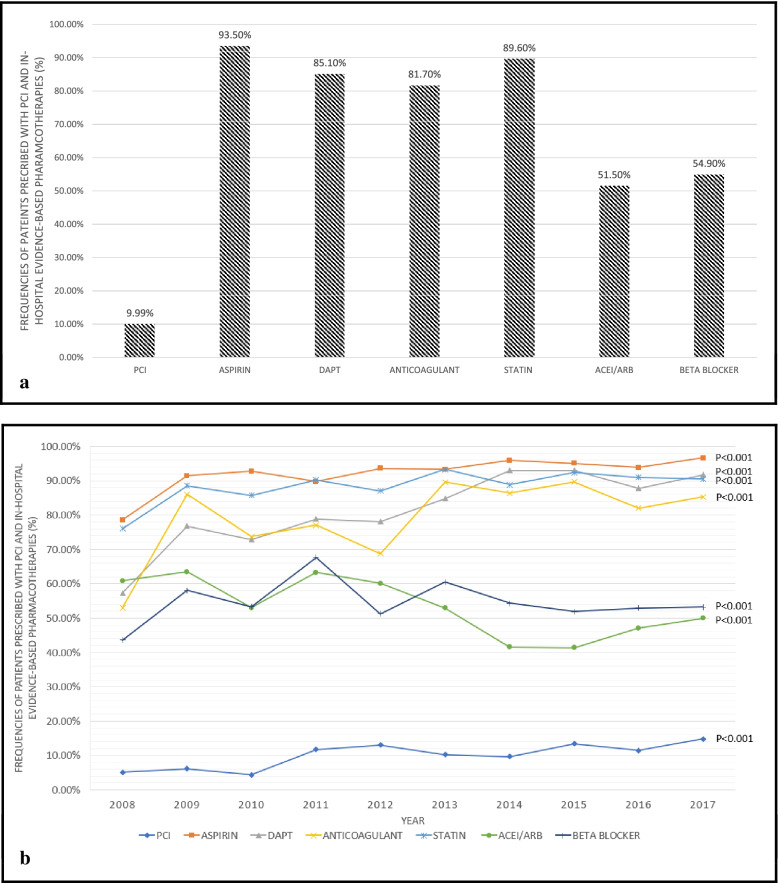
**a**: 10-years cumulative frequencies of PCI and in-hospital evidence-based pharmacotherapies for octogenarians with ACS in the NCVD-ACS registry (2008–2017). In-hospital pharmacotherapies include aspirin (monotherapy or as combined therapy), dual antiplatelet therapy (DAPT), anticoagulants, statins, ACE inhibitors(ACEIs)/angiotensin II receptor blockers (ARBs) and beta- blockers are individually illustrated. **b**: Frequencies of PCI and in-hospital evidence-based pharmacotherapies prescribed to octogenarians with ACS in the Malaysian NCVD-ACS registry (2008–2017). In-hospital pharmacotherapies include aspirin (monotherapy or as combined therapy), dual antiplatelet therapy (DAPT), anticoagulants, statins, ACE inhibitors(ACEIs)/angiotensin II receptor blockers (ARBs) and beta- blockers. Linear trend test was used to determine the *P*-values. Frequencies of PCI and in-hospital evidence-based pharmacotherapies in octogenarians with ACS in the NCVD-ACS registry (2008–2017)

Cumulatively, aspirin (93.5%), DAPT (85.1%), anticoagulants (81.7%) and statins (89.6%) were prescribed at near maximal levels, whilst about half were prescribed ACEIs/ARBs (51.5%) and beta-blockers (54.9%) in the 10-years as presented in Fig. 2a. The in-hospital pharmacotherapies frequencies for the respective studied years are summarised in Fig. 2b. An increased rate of octogenarians treated with PCI was observed, from 5.1% in 2008 to 14.8% in 2017 (Fig. 2b). In general, the prescribing of evidence-based pharmacotherapies has increased over the 10 years (*p* < 0.001).

PCI intervention was performed at greater frequencies than CABG in all ACS stratum, (Table 2). Less than 13% of STEMI patients had undergone primary PCI, while 60% received intravenous thrombolysis. There were significant differences in the prescribing of aspirin, DAPT, anticoagulants, statins, ACEIs/ARBs and beta-blockers when comparing the ACS stratum. Octogenarian women with ACS were less likely to undergo PCI than men with an aOR of 0.698 (95% CI 0.490–0.993), as shown in Table 3. NSTEMI and UA were less likely to receive PCI but more likely given anticoagulants than STEMI. NSTEMI octogenarians were also more likely to be prescribed with DAPT, while those with UA were more likely to be prescribed ACEIs/ARBs, and beta-blockers than STEMI. Octogenarians with known congestive heart failure were more likely to receive PCI and prescribed statins, ACEIs/ARBs and beta-blockers than those without. Those with hypertension and previous history of IHD were more likely to be prescribed ACEIs/ARBs than those without these CV risk factors. Those with co-morbidity of CKD were less likely to be prescribed beta-blockers than those without CKD.

**Table 2:**
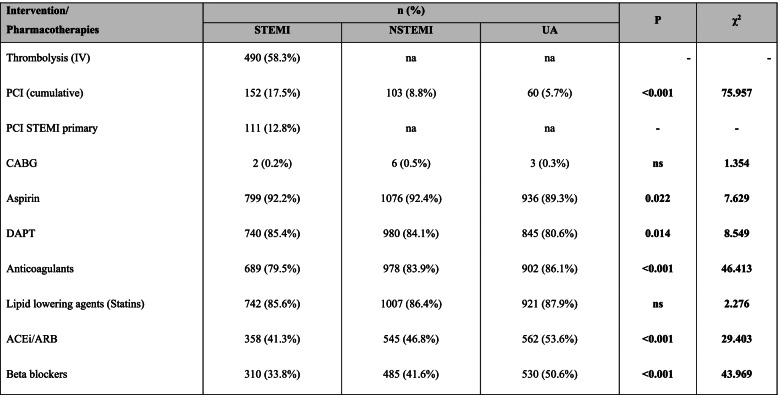
Characteristics of in-hospital intervention (PCI) and evidence-based pharmacotherapies for octogenarian from 2008 to 2017 NCVD-ACS registry, comparing the ACS stratum

χ^2^: chi-square value (Pearson). P: probability value. na: not applicable. ns: not significant (*p* > 0.05)

**Table 3:**
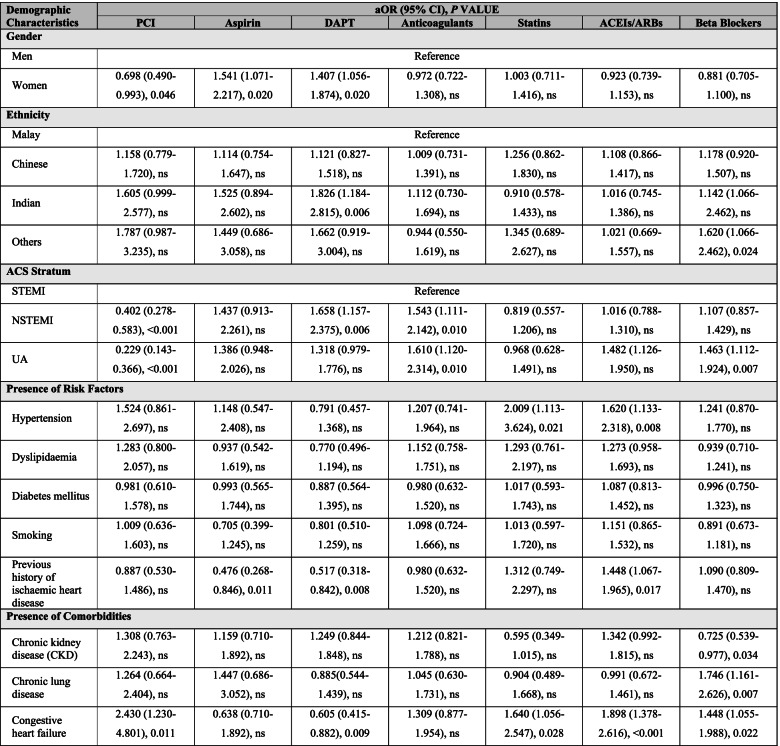
Variations in the in-hospital PCI and evidence-based pharmacotherapies for Malaysian octogenarians with ACS, presented as adjusted OR and 95%CI

aOR (adjusted Odds Ratio) and 95% CI (confidence interval) was calculated using binary logistic regression

The OR and 95% CI for intervention and in-hospital pharmacotherapies were adjusted for gender, ethnicities, types of ACS, risk factors and comorbidities

Reference: Gender1 men; ethnicity2 Malay; types of ACS3 STEMI; cumulative risk factors41/5

For risk factor and comorbidities, the references were those without the respective risk factors and comorbidities

Excluded from analysis due to low number of exposures: Intervention- CABG; Co-morbidities – cerebrovascular and vascular

*NC: Not computed. Data was not computed due to low number of participants

ns: not significant (p > 0.05)

## Discussion

This study found an increasing trend of octogenarians with ACS in this multi-ethnic country, with NSTEMI presentation predominated. The Swedish National registry reported that the cumulative incidence of MI for those aged 80 years old and above are the highest amongst other age groups [23]. Thus, octogenarians represent an important group for policymakers to focus on in the coming years. A study on nonagenarians in Poland showed a higher risk of periprocedural adverse outcomes than younger patients [24]. Thus, it would be interesting to examine octogenarians, nonagenarians and centenarians separately. However, patients aged 80 years old and beyond made up only 5% of the total ACS population in this country.

In contrast to the ACS trends in the younger age group [16, 25], the frequencies of men and women did not differ in the octogenarians. However, women had higher rates of hypertension, DM and dyslipidaemia, while men had higher smokers and IHD, similar to the Malaysian National Health and Morbidity Survey (NHMS) for adults in 2015 [26]. The largest ethnic group were the Chinese, in contrast to the ethnic distribution in the country, where Malays are the majority [27]. This ethnic variation also differed from the ACS trend in younger groups [16]. The Chinese predominance in this age group could be due to several reasons, such as longer lifespan, healthier lifestyles and geographical location. They have the lowest prevalence of CV risk factors and physical inactivity than other ethnicities [28, 29] and reside primarily in urban areas where healthcare services are nearby [30].

There is a high prevalence of octogenarians with multiple CV risk factors, affecting the presentation, severity, and complexity of managing ACS. Hypertension remains the major risk factor. Thus, advocating evidence-based management to treat hypertension may result in a more favourable outcome [31]. More than 40 % of patients have DM, which is higher than observed in the African, European, Northern America and the Western Pacific region [32]. DM is associated with complications such as CKD and needs to be managed optimally in this group of patients. Comorbidities such as congestive heart failure have also influenced the choice of management of ACS in these patients.

There was an increasing trend of PCI in octogenarians with STEMI, despite the rates being relatively low compared to what is being practised in other countries [33]. The advantages of PCI are still debatable in old age. Japan, a country with the longest life expectancy globally, reported a 3-fold higher in-hospital survival in octogenarians who underwent PCI using the Japan Acute Myocardial Infarction Registry (JAMIR) [34]. The most common non-cardiac complication of PCI is bleeding, associated with a higher risk of death in the elderly [35]. The rate of PCI will likely continue to increase in the coming years; therefore, skilled cardiologists and equipped facilities are essentials. CABG was not preferred in our population, most likely influenced by their frailties and comorbidities [36]. The treatments for NSTEMI and UA are mainly through pharmacotherapies, focusing on antiplatelets and anticoagulants [37]. This practice is reflected in this study, whereby they were more likely to receive anticoagulants than STEMI. Interestingly, the ‘After Eighty’ study has suggested that participants with NSTEMI and UA may have better outcomes, with higher success rates and less complication after PCI than those treated with optimum medical treatment alone [38].

The prescribing of pharmacotherapies in the octogenarians with ACS were in accordance with international standard [37]. Recent ACS guidelines have recommended aspirin as part of DAPT [37] and shown to benefit the older age group [39]. This is reflected in this study, whereby the prescribing of DAPT has increased. Statins are recommended in the elderly, taking into consideration the pharmacokinetics of each statin [40]. The recommendations for ACEIs/ARB for ACS in the guideline is similar to those of statins [20]. However, since the initial time point, the prescription of ACEIs/ARBs showed the opposite trend to statins. The underutilisation of ACEIs/ARBs was also seen in developed countries such as the US despite guidelines [40, 41]. A similar trend was observed with beta-blockers. Clinical preference of physicians may influence the ‘hesitancy’ in using ACEIs/ARBs and beta-blockers and need to be investigated further. Nonetheless, similar trends were seen in the younger age groups in the NCVD-ACS registry [15, 16].

We need to bridge the knowledge gap on the effectiveness and risks of current management by performing clinical studies in the local setting. Physicians need to be aware of age-related physiological changes that may affect the pharmacokinetics and pharmacodynamics of these drugs in octogenarians while considering the immediate and long-term benefits. An example that could be modelled is Finland’s follow-up accumulation of risk factor study [42] and the After Eighty case-control study. A CV modelling study should be performed to investigate the contributions of each risk factor and how management impacted the outcomes in this group of patients. One example is the IMPACT-CHD model used in the Italian and Portuguese population that have shown death prevented and postponed in women aged ≥75 were higher than men [43, 44].

### Strengths and limitations

This study uses data from a nationwide cardiology registry obtained from 24 voluntary hospitals to represent an unselected group of octogenarian patients with ACS in a real-world setting in this country. The database is well maintained, and training is provided regularly for those involved to ensure data quality [45]. A one-time data capturing system ensures no replication of patients within the registry [17].

This registry only captures data of those reported to the participating hospitals; thus there may be a selection bias. Information that measures socioeconomic status that may influence demographics presentation, such as occupation and educational levels, were also unavailable. Elderly patients have varied tolerability towards intervention and pharmacotherapies [38]. The spectrum and complexity of the disease and its management require a detailed investigation of the patients’ clinical characteristics. Data on biochemical profiles, the dosage of prescriptions, concurrent medications, and detailed clinical information such as stages of CKD were not available. This study did not delve into the PCI’s technicalities and clinical practice, such as the type of stent used, the cardiac catheterisations site of access, either radial or femoral approach and the cardiologist experience.

The variations in PCI and evidence-based pharmacotherapies may impact short- and long-term outcomes in this group of patients [46, 47]. This study focused on the in-hospital management of ACS in broad consecutive time points. The reported trends of intervention and in-hospital prescription in the elderly would serve as valuable information for the policymakers and hospital administrators in terms of management of elderly with ACS. Future studies should include outcomes such as in-hospital and 1-year mortality, readmission and re-PCI.

## Conclusion

As octogenarians with ACS continue to increase, the country needs to plan for disease management and prevention. Despite being the most vulnerable age group, octogenarians were not receiving aggressive treatment as younger patients. The use of evidence-based pharmacotherapies was maximised instead. It is expected that ACS management will continue to improve with an increasing number of octogenarians, better technologies, enhanced cardiologist skills and knowledge of disease in this age group.

## Supplementary Information

**Additional file 1.**

## Data Availability

All data generated or analysed during this study are included in this published article.
