# Supplementary material for: Intervention and in-hospital pharmacoterapies in octogenarian with acute coronary syndrome: a 10-year retrospective analysis of the Malaysian National Cardiovascular Database (NCVD) registry
Source: BMC Geriatr. 2022 Jan 4;22:23. doi: 10.1186/s12877-021-02724-7 (PMC8729007; doi:10.1186/s12877-021-02724-7)
Supplement: Supplementary file 1 — Additional file 1. [file 12877_2021_2724_MOESM1_ESM.pdf]

Supplementary table 1 Clinical characteristics of Malaysian octogenarians with ACS from 2008-2017 in the NCVD-ACS registry.

Clinical characteristics	Year											
	**2008** n=117	**2009** n=179	**2010** n=158	**2011** n=268	**2012** n=350	**2013** n=374	**2014** n=378	**2015** n=390	**2016** n=403	**2017** n=545	**Total** n=3,162	**P**
Presence of risk factors												
Hypertension	74.20%	83.50%	83.20%	80.90%	85.30%	79.90%	80.30%	83.10%	86.20%	81.40%	82.30%	ns
Dyslipidaemia	33.30%	37.10%	40.80%	34.90%	46.00%	48.10%	41.60%	47.30%	50.70%	46.00%	44.60%	0.002
Diabetes mellitus	47.00%	47.40%	54.70%	46.00%	52.60%	41.20%	47.40%	48.20%	50.80%	44.00%	47.40%	ns
Smoking	34.70%	35.20%	37.00%	26.90%	35.50%	34.30%	31.50%	32.90%	30.40%	32.00%	32.60%	ns
Previous history of Iischaemic heart disease	3.00%	11.30%	11.60%	9.50%	7.60%	6.00%	7.00%	4.40%	5.20%	6.20%	6.70%	ns
Presence of comorbidities												
Chronic kidney disease	12.50%	15.00%	20.80%	11.40%	18.10%	15.70%	14.30%	15.50%	15.80%	15.40%	15.50%	ns
Chronic lung disease	10.10%	10.30%	10.60%	7.80%	5.00%	8.70%	6.90%	7.30%	8.70%	7.60%	7.80%	ns
Congestive heart failure	12.20%	15.10%	16.70%	20.50%	18.30%	12.30%	11.50%	11.70%	10.50%	10.60%	13.30%	0.001
Cerebrovascular disease	5.10%	5.00%	7.80%	7.30%	7.60%	6.90%	9.50%	4.90%	6.00%	5.50%	6.60%	ns
Peripheral vascular disease	0.00%	0.00%	1.60%	2.50%	0.00%	0.00%	0.60%	2.00%	0.80%	0.80%	0.90%	0.012
Killip score*												
Class I	51.20%	65.60%	53.80%	50.30%	48.50%	55.60%	57.90%	55.20%	55.40%	57.60%	55.40%	0.002
Class II	32.50%	23.20%	33.10%	34.60%	35.50%	29.70%	21.50%	20.30%	25.20%	24.70%	26.80%	
Class III	6.30%	5.30%	7.70%	7.30%	6.10%	7.50%	9.80%	12.10%	10.10%	6.70%	8.30%	
Class IV	10.00%	6.00%	5.40%	7.80%	10.00%	7.10%	10.80%	12.40%	9.40%	11.00%	9.50%	
Cardiogenic shock	10.00%	6.00%	5.40%	7.80%	10.00%	7.10%	10.80%	12.40%	9.40%	11.00%	9.50%	<0.001

Killip Score*: Values counted from patients with STEMI and NSTEMI only. P: probability value. ns: not significant ($p>0.05$). n: number of patients.
